# Author Correction: The dynamics of mutational selection in cutaneous squamous carcinogenesis

**DOI:** 10.1038/s42003-026-10228-6

**Published:** 2026-05-21

**Authors:** Greta Skrupskelyte, Joanna C. Fowler, Stefan Dentro, Carine Winkler, Irina Abnizova, Niklas Beumer, Roshan Sood, Thomas Quarrell, Charlotte King, Jivko Kamarashev, Emmanuella Guenova, Moritz Gerstung, Benjamin A. Hall, Liliane Michalik, Philip H. Jones

**Affiliations:** 1 Cambridge Stem cell Institute, University of Cambridge, Cambridge, UK; 2 Wellcome Sanger Institute, Wellcome Genome Campus, Hinxton, Cambridge, UK; 3 European Molecular Biology Laboratory, European Bioinformatics Institute, Cambridge, UK; 4 Artificial Intelligence in Oncology (B450), Deutsches Krebsforschungszentrum, Heidelberg, Germany; 5 Centre for Integrative Genomics, Faculty of Biology and Medicine, University of Lausanne, Lausanne, Switzerland; 6 Institute of Immunology, University Medical Centre Mainz, Mainz, Germany; 7 Research Centre for Immunotherapy, University Medical Centre Mainz, Mainz, Germany; 8 School of Clinical Medicine, Addenbrooke’s Hospital, Hills Rd, Cambridge, UK; 9 Department of Dermatology, UniversitätsSpital, Zurich, Switzerland; 10 Clinical Department of Immunodermatology, Kepler University Clinic and Medical Faculty, Johannes Kepler University, Mainz, Austria; 11 Dept of Med Physics & Biomedical Engineering, University College London, London, UK; 12 Clinical Research Institute for Inflammation Medicine, Medical Faculty, Johannes Kepler University, Linz, Austria; 13 Department of Oncology, University of Cambridge, Hutchinson Research Centre, Cambridge Biomedical Campus, Cambridge, UK

**Keywords:** Cancer genetics, Cancer genomics

Correction to: *Communications Biology* 10.1038/s42003-025-09406-9, published online 12 January 2026

“In this article the author name Niklas Beumer was incorrectly written as Niklaus Beumer. The original article has been corrected.”

