# Supplementary material for: The dynamics of mutational selection in cutaneous squamous carcinogenesis
Source: Commun Biol. 2026 Jan 12;9:127. doi: 10.1038/s42003-025-09406-9 (PMC12855962; doi:10.1038/s42003-025-09406-9)
Supplement: Supplementary file 2 — Supplementary Material [file 42003_2025_9406_MOESM2_ESM.pdf]

Supplementary Material

The dynamics of mutational selection in cutaneous squamous carcinogenesis

Supplementary Table 1

Supplementary Figures 1-9

Weeks UV	0	8	16	18	20	21	22	23	24	27
Number of mice treated	6	10	4	2	1	4	1	2	18	4
Number of mice sequenced (epidermis)	6	4	4	2	0	1	0	1	4	4
Number tumours total	0	0	6	5	1	10	5	9	84	30
Number tumours sequenced (number of mice)	0	0	6(4)	5(2)	0	1(1)	5(1)	6(1)	28(4)	28(4)

Supplementary Table 1: Numbers of mice, epidermal and tumors sequenced at each time point.

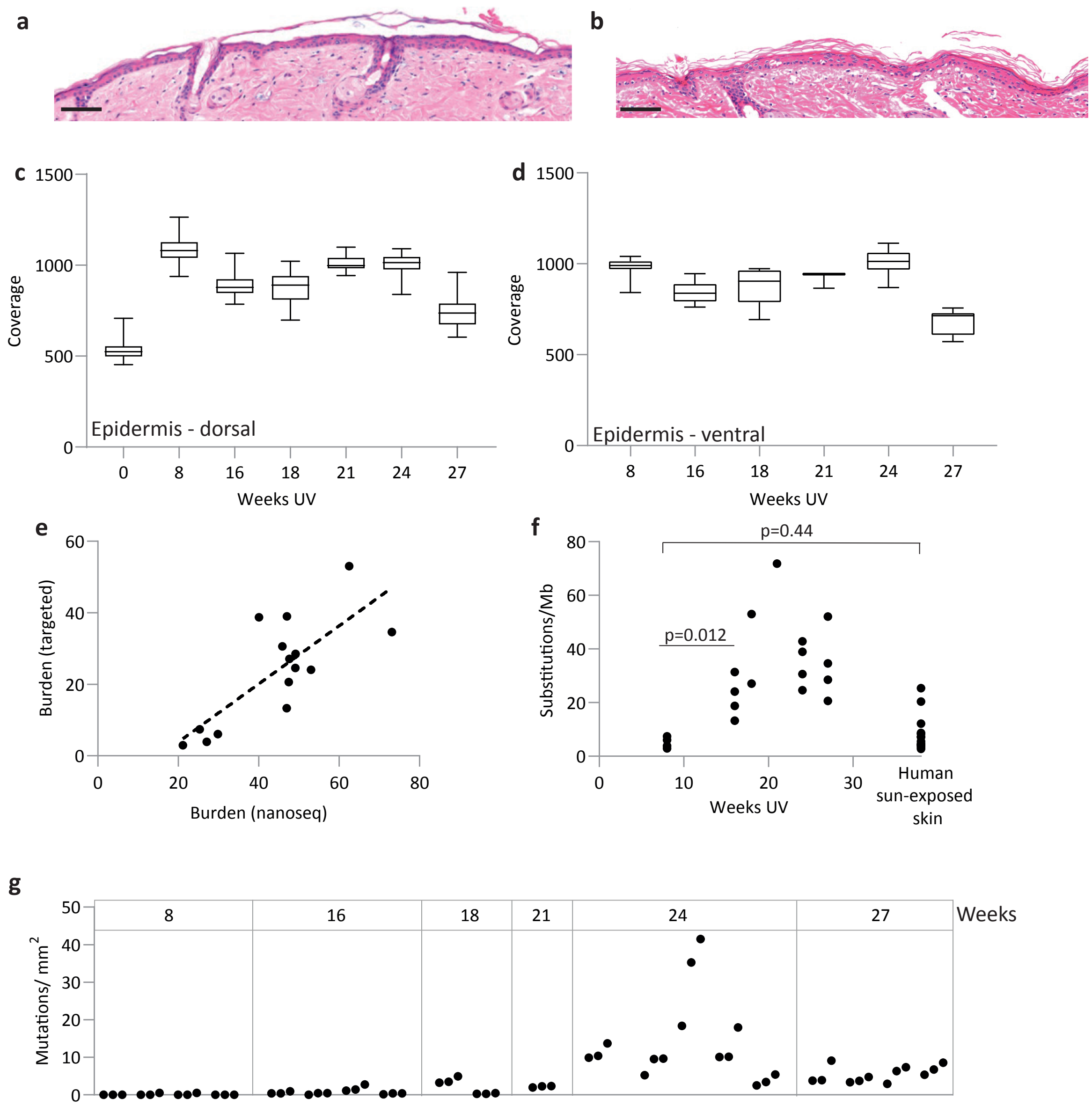

Supplementary Figure 1: UV irradiation and mutation in epidermis. **a,b** Histology of dorsal skin pre (a) and post (b) UV exposure for 8 weeks. Scale bar = 100µm **c,d** Sequencing coverage from dorsal and ventral epidermis. Boxes indicate median and 25th and 75th percentiles, bars maximum and minimum values. Source Data: Supplementary Data 2. **e** Correlation of mean mutational burden (SBS/Mega base) in epidermis estimated by NanoSeq or targeted sequencing. Line shows linear regression, $r^2 = 0.6$, p=0.0007, n=15 mice Source Data: Supplementary Data 3 and 5. **f** Mutational burden in dorsal epidermis estimated from targeted sequencing, Human sun exposed skin, mutational burden estimated from targeted sequencing[1]. P-value derived from Kruskal-Wallis test. Source data: Supplementary Data 5. **g** Mutations/mm^2 in ventral epidermis, each dot is a sample, samples grouped by mouse. Source data: Supplementary Data 5.

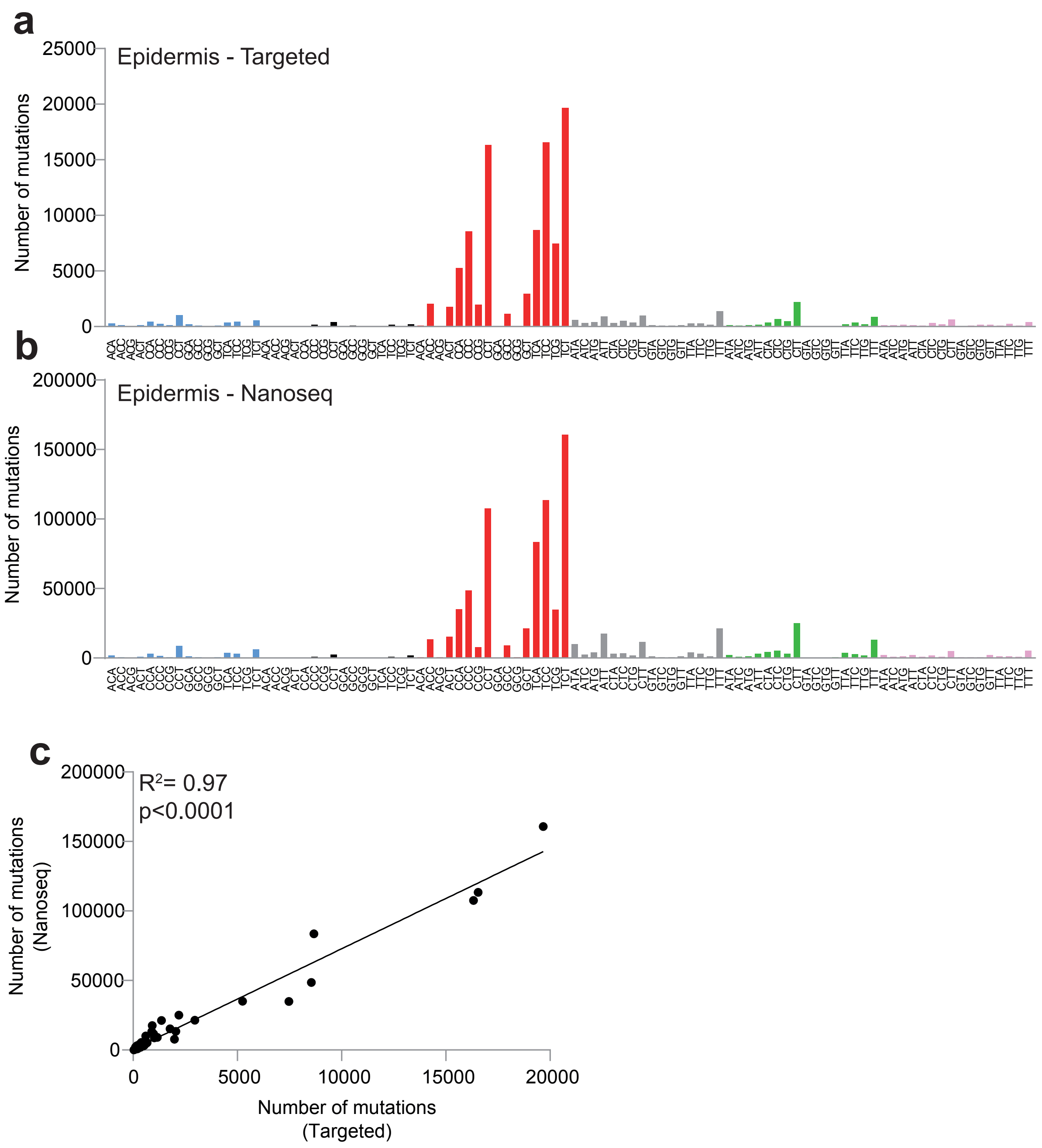

Supplementary Figure 2. Mutational spectra of targeted and nanoseq epidermal sequencing. a, **b**. Mutational spectra of targeted sequencing (**a**) and Nanoseq (**b**). Source data: Supplementary Data 4. **c**, Correlation of spectra shown in **a** and **b**, each dot represents a trinucleotide context, Pearson's test.

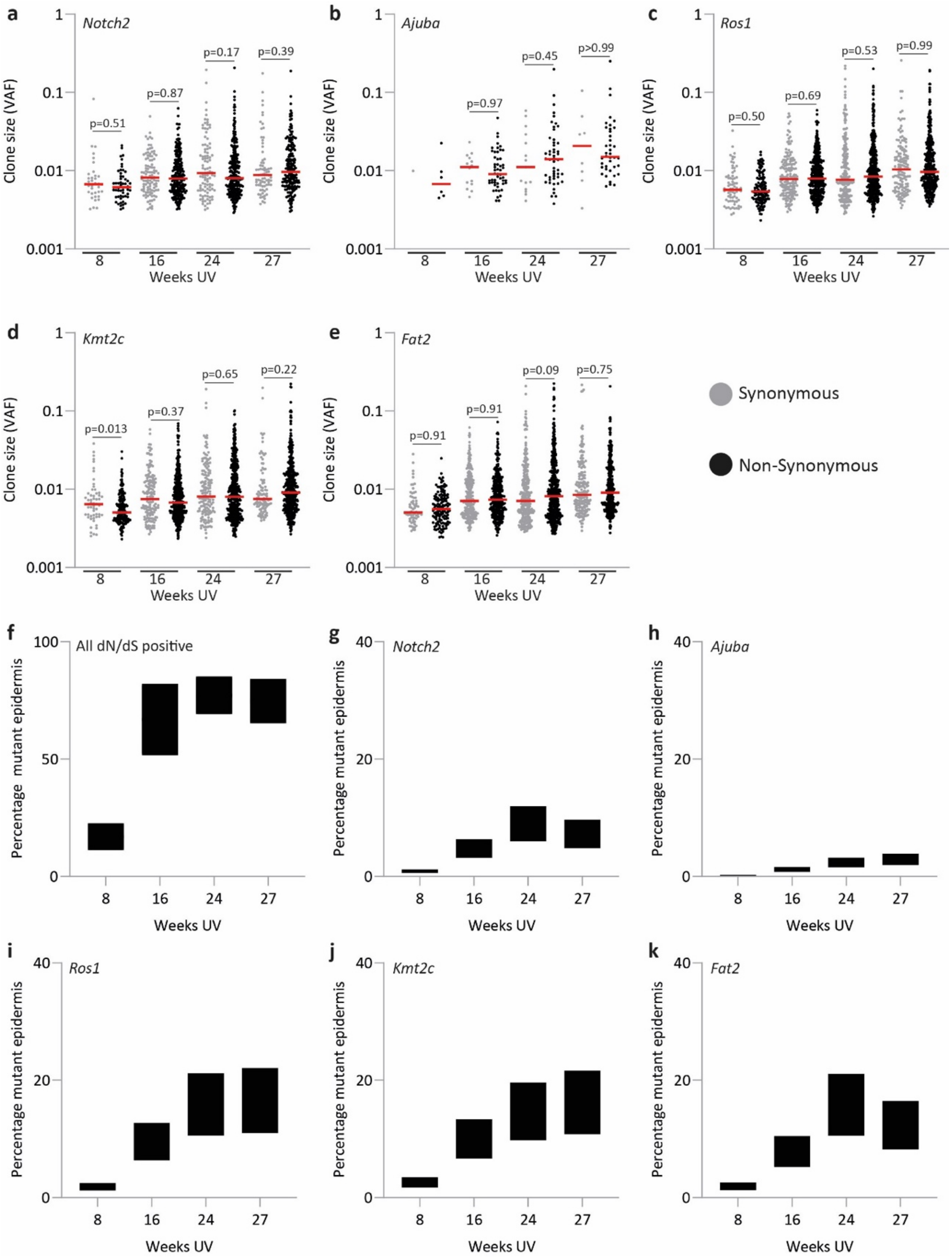

Supplementary Figure 3: Vaf_ns/Vaf_s and proportion of mutant epidermis for genes under positive selection a-e VAF distribution of nonsynonymous (black points) and synonymous (grey points) for *Notch2* (**a**), *Ajuba* (**b**), *Ros1* (**c**), *Kmt2c* (**d**), and *Fat2* (**e**). Red lines indicate median, 2-tailed Wilcoxon test. Source data: Supplementary Data 5. **f-k** Percentage area of dorsal epidermis colonized by nonsynonymous mutants of all positively selected genes by dN/dS (**f**), and *Notch2* (**g**), *Ajuba* (**h**), *Ros1* (**i**), *Kmt2c* (**j**) and *Fat2* (**k**), estimated from summed variant allele frequency at each time point. Data shows average across all mice for each time point, n=4 mice for each time point. Upper and lower bounds indicate uncertainty in copy number and multiple mutations per cell. Source data: Supplementary Data 8.

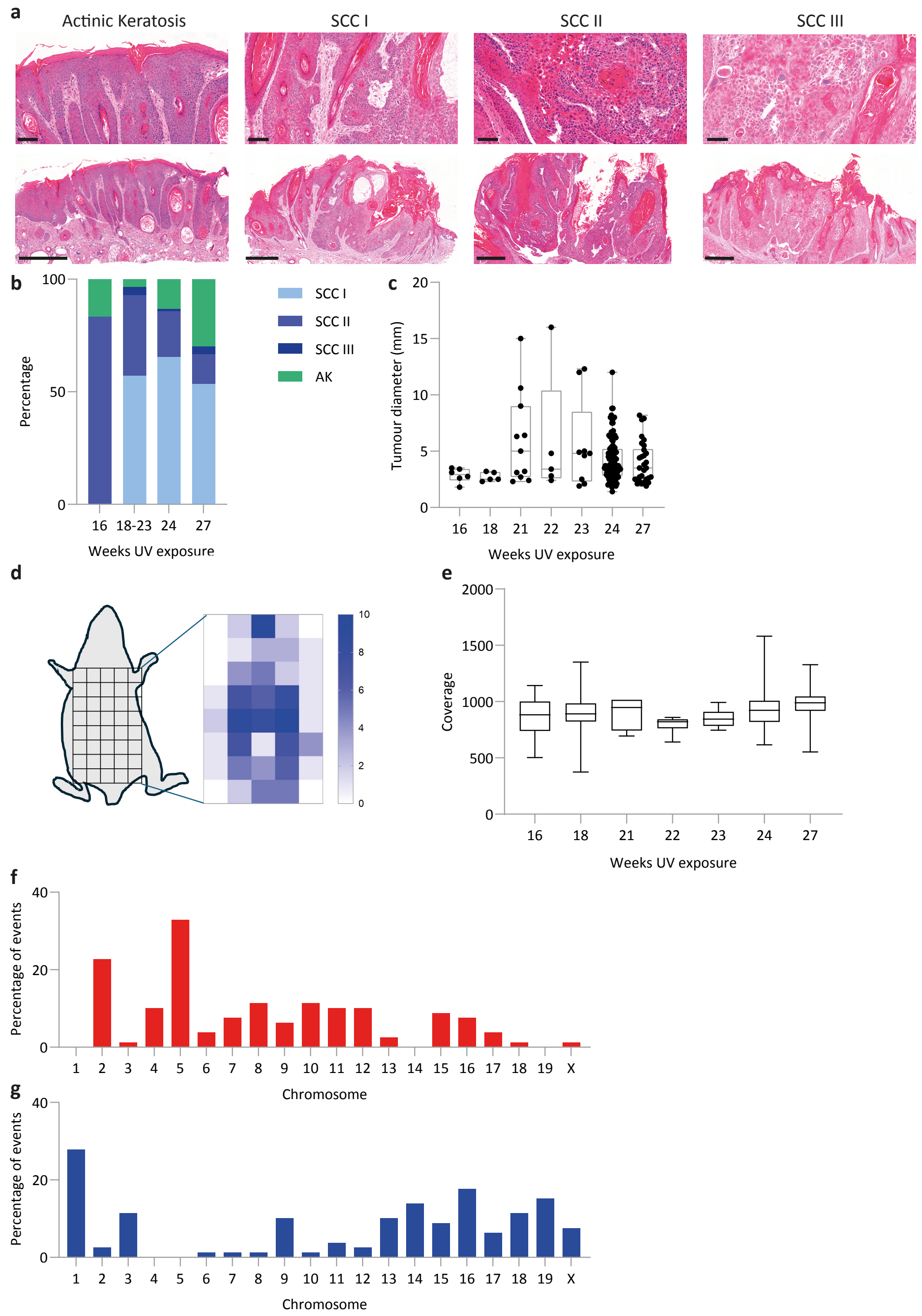

Supplementary Figure 4: Tumor characteristics a, Representative histological images of all tumor grades observed. 40x (top panels scale bar=100µm) and 10x (bottom panels scale bar=500µm) magnification **b,** Histological tumor grades observed at each time point (n tumors:16 weeks, 6; 18 weeks, 5; 20 weeks, 1; 21 weeks 8; 22 weeks 5; 23 weeks 9; 24 weeks 84; 27 weeks 30). **c,** Tumor diameter (mm) at each time point. Each point represents a tumor. **d,** Heatmap of tumor locations in dorsal skin. Source data: Supplementary Data 9. **e,** Targeted sequencing coverage across tumor slices. Source Data: Supplementary Data 2. **f-g**, Overview of distribution of copy number changes across all chromosomes. CNA calls are pooled across

all timepoints. Distribution of gains (**f**) and losses (**g**) are shown. Source Data: Supplementary Data 11.

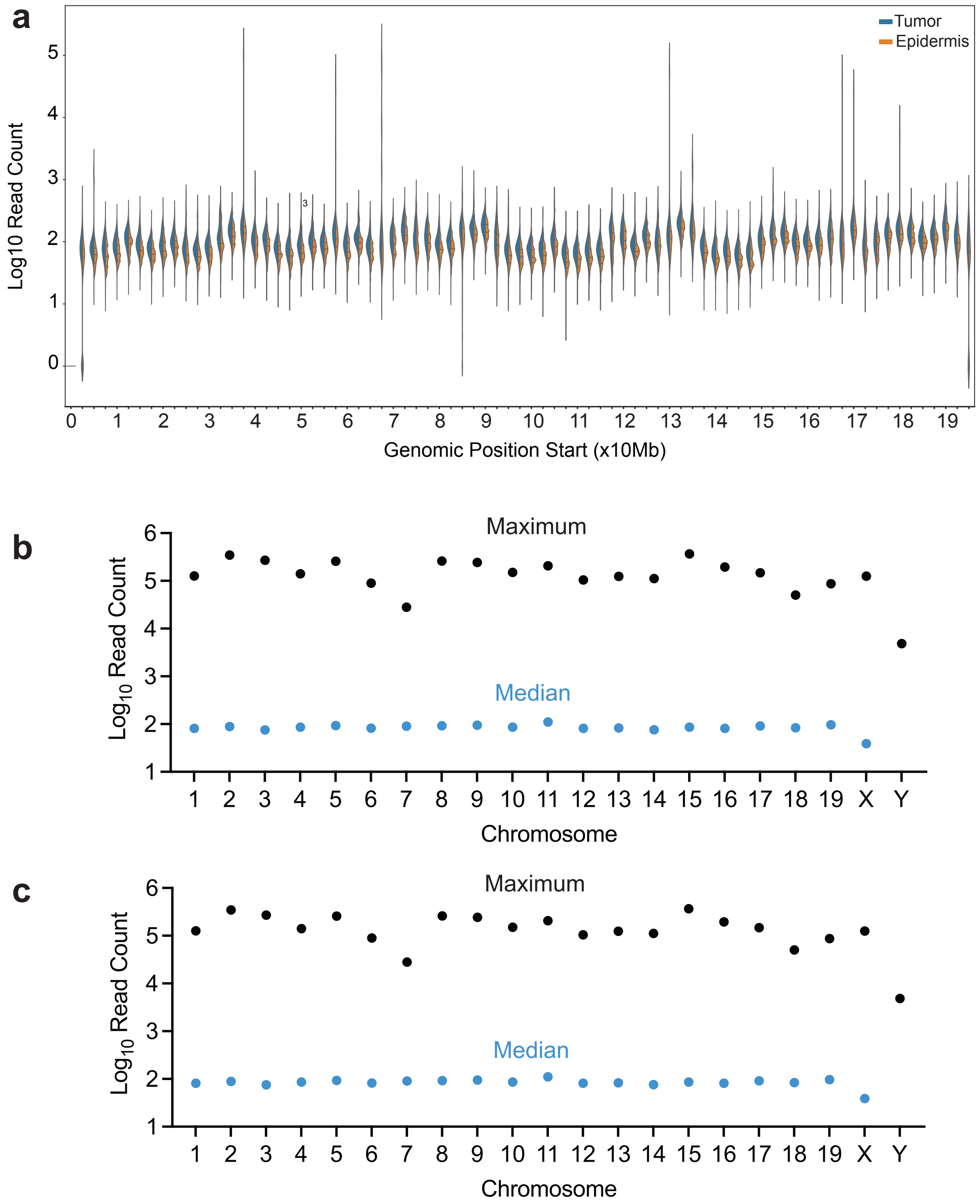

Supplementary Fig. 5, Copy number analysis using off target reads. a, Read count of off target reads mapped to Chromosome 1, with genomic start position analysed in 2.5 Mega base intervals, tumors in blue. epidermis in orange. Plots for all chromosomes available on Figshare (https://doi.org/10.6084/m9.figshare.30246841.v3). **b, c,** Dots show median (blue) and maximum (black) read counts by chromosome in epidermis (**b**) and tumors (**c**). The minimum read count was zero for all chromosomes. Source Data: BAM files from ENA ERP166414 processed using the pipeline in [2].

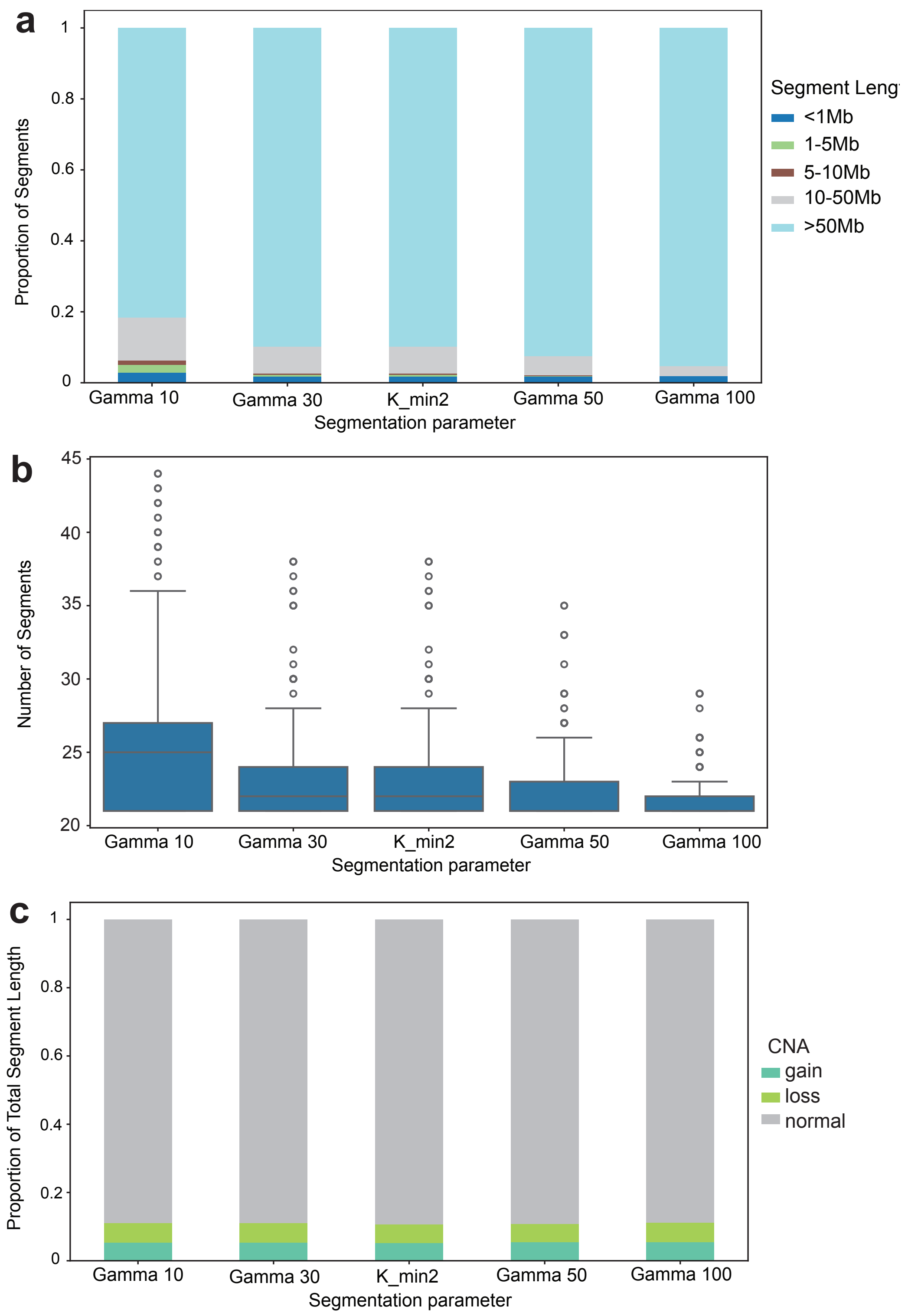

Supplementary Figure 6: **Copy number with off target reads**: Results of parameter sweep of segmentations settings in copy number calling algorithm, see **methods** for details. **a**, Segment length distribution by segmentation parameter. **b**, number of segments by segmentation parameter. **c**, Proportion of CNA alterations by segmentation parameter. Source Data: Source Data: BAM files from ENA ERP166414 processed using the pipeline in [2].

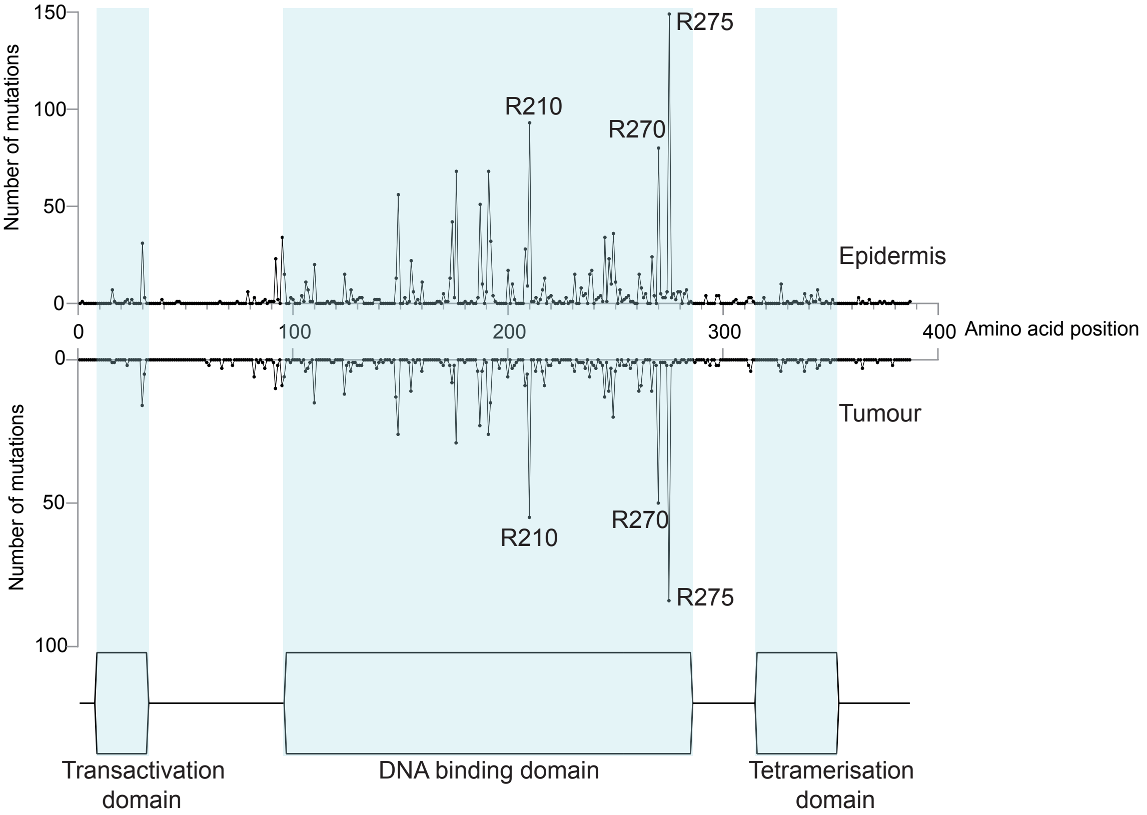

Supplementary Fig. 7: Distribution of *Trp53* missense mutations in epidermis (upper panel) and tumors (lower panel). Source Data: Supplementary Data 5 and 10.

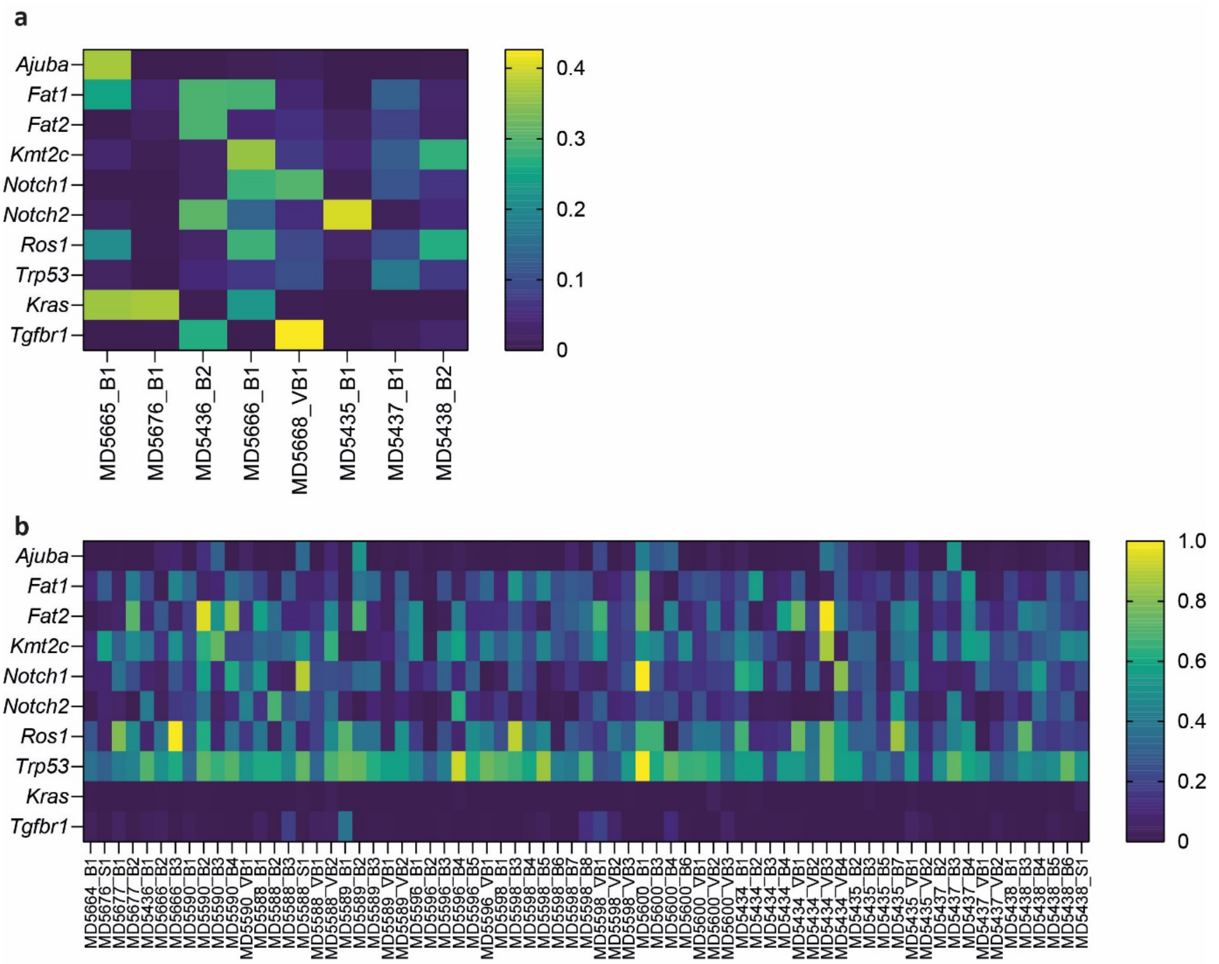

Supplementary Figure 8: Heatmaps showing the average summed VAF per tumor in mutant *Trp53*-low (**a**) and *Trp53*-high tumors (**b**). Only genes under positive dN/dS selection are shown. *Kras* mutations were gain of function (G12C in MD5665_B1, G12D in MD5676_B1 and P34L in MD5666_B1). Source Data: Supplementary Data 10.

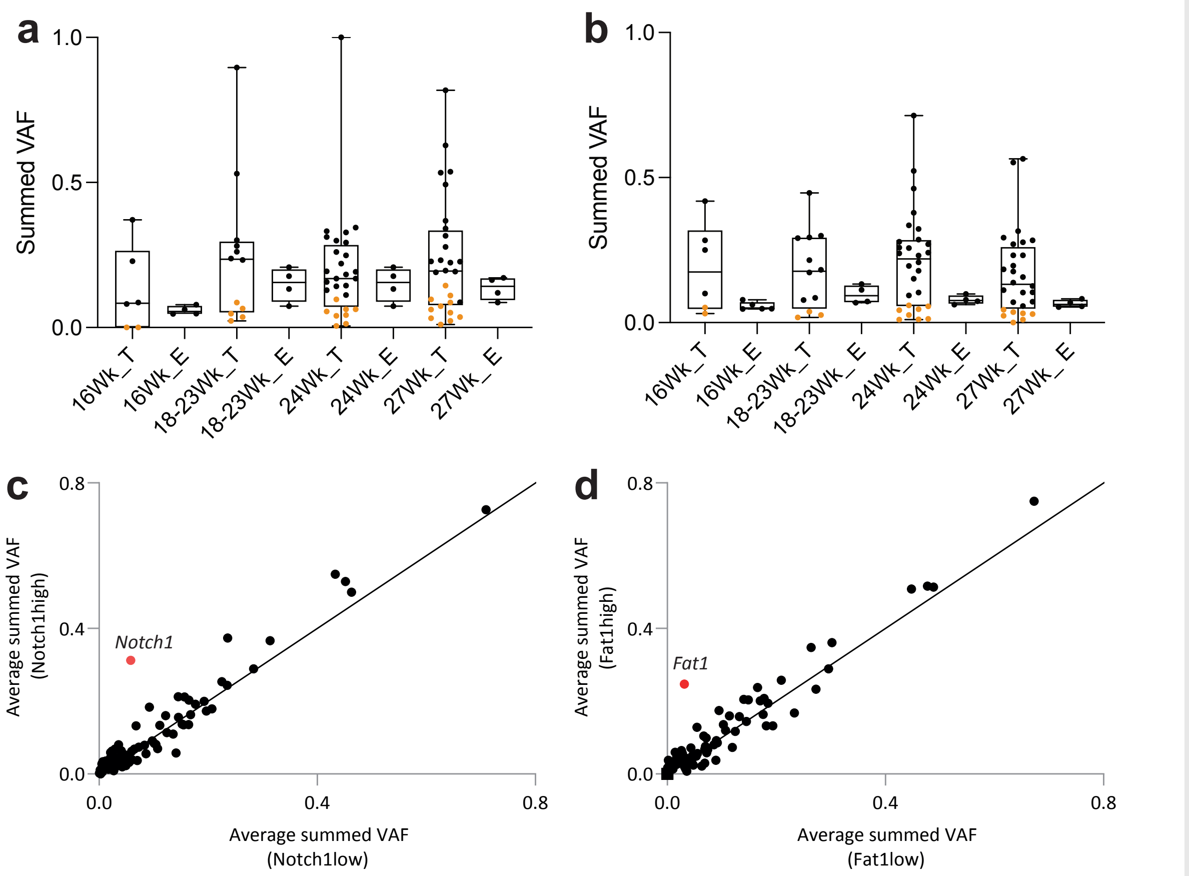

Supplementary Figure 9: Mutational selection in tumors varying in *Notch1* and *Fat1* mutations. **a, b** Fraction of tissue (summed VAF) with nonsynonymous mutants of *Notch1,* **a,** and *Fat1,* **b**. Dots are tumors (T) or epidermis (E), Tumors in orange have a lower proportion of mutant *Notch1 or Fat1* than epidermis in the same animal. Source Data: Supplementary Data 15. Central bar is median, box indicates quartiles, bars indicate range. Comparison of summed VAF in epidermis and tumour p=4.8×10^{-16} , 2 tailed nested Anova. **c,d** Selection in mutant-high and-low tumors, **c**, *Notch1*, **d**, *Fat1*. Summed VAF (proportion of mutant tissue) is plotted for each sequenced gene, each dot is a gene, line indicates equivalence in both groups. 2 tailed Z-test with Benjamini-Hochberg multiple test correction for outliers. Orange, significantly different mutant genes, *Notch1* p= 1.2×10^{-11}, *Fat1* p= 9.0×10^{-11}. Source Data: Supplementary Data 15.

Supplementary References

1. Fowler JC*, et al.* Selection of Oncogenic Mutant Clones in Normal Human Skin Varies with Body Site. *Cancer Discovery* **11**, 340-361 (2021).

2. Sood R, Jones PH. Skrupskelyte etal SI code: Publication supporting code and notebooks.) (2025).
