# Supplementary material for: The dynamics of mutational selection in cutaneous squamous carcinogenesis
Source: Commun Biol. 2026 Jan 12;9:127. doi: 10.1038/s42003-025-09406-9 (PMC12855962; doi:10.1038/s42003-025-09406-9)
Supplement: Supplementary file 3 — Description of Additional Supplementary Files [file 42003_2025_9406_MOESM3_ESM.pdf]

Description of Additional Supplementary Files:

Supplementary Data 1: Gene bait list

Supplementary Data 2: Targeted sequencing coverage

Supplementary Data 3: Mutational burden from Nanoseq

Supplementary Data 4: Mutation Spectrum, targeted sequencing and Nanoseq

Supplementary Data 5: Mutations called from normal epidermal sequencing

Supplementary Data 6: dN/dS from normal epidermal sequencing

Supplementary Data 7: Median synonymous and non-synonymous clone size in epidermis

Supplementary Data 8: Percentage mutant epithelium of genes under positive selection

Supplementary Data 9: Overview of Tumor sequencing

Supplementary Data 10: Mutations called from tumor sequencing

Supplementary Data 11: Copy Number Calls for tumor sections

Supplementary Data 12: dN/dS from tumour sequencing

Supplementary Data 13: Median synonymous and non-synonymous clone size in tumours

Supplementary Data 14:Percentage mutant tumour of genes under positive selection

Supplementary Data 15: Comparison of % mutant tissue in between tumor and epidermis
